# Effect of Surgically Induced Weight Loss on Pelvic Organ Prolapse: A Meta-analysis

**DOI:** 10.1007/s11695-023-06867-x

**Published:** 2023-10-07

**Authors:** Zhao Tian, Xiuqi Wang, Xiaopeng Hu, Zhijing Sun

**Affiliations:** 1 Department of Obstetrics and Gynecology, Peking Union Medical College Hospital, Peking Union Medical College, Chinese Academy of Medical Sciences, National Clinical Research Center for Obstetric & Gynecologic Diseases, Beijing, China; 2 Department of Structural Heart Disease, National Center for Cardiovascular Disease, China & Fuwai Hospital, Chinese Academy of Medical Sciences & Peking Union Medical College, Beijing, China

**Keywords:** Bariatric surgery, Pelvic organ prolapse Symptoms, Pelvic Organ Prolapse Distress Inventory

## Abstract

**Introduction:**

Bariatric surgery alleviates certain aspects of pelvic floor disorder, but the effect on pelvic organ prolapse (POP) is unclear. To assess the effect of bariatric surgery on POP we conducted the present meta-analysis and firstly performed a subgroup analysis based on the duration of follow-up.

**Methods:**

Four databases including PubMed, The Cochrane Library, Web of Science, and Embase were searched to identify relevant studies published before February 24, 2023. The main outcome was the prevalence and severity of POP symptoms before and after bariatric surgery. Then we assessed the heterogeneity, publication bias and performed subgroup analyses based on follow-up time, study quality and region.

**Results:**

Eleven studies with a total of 696 participants met the inclusion criteria. The results showed that the prevalence of POP decreased after bariatric surgery (odds ratio[OR] = 2.29, 95% confidence interval[CI]: 1.05, 5.01; P = 0.04, I^2^ = 78%), with significant differences observed both at 3–6 months (OR = 2.24, 95% CI: 1.25, 4.01; P = 0.007, I^2^ = 59%) and 12 months (OR = 4.64, 95% CI: 2.83, 7.58; P < 0.0001, I^2^ = 0%) of follow-up compared with pre-surgery. Pelvic Organ Prolapse Distress Inventory scores 6-item also decreased after bariatric surgery (mean difference [MD] = 2.11, 95% CI: 0.32, 3.89; P = 0.02, I^2^ = 55%) with significant differences observed both at 3–6 months (MD = 3.72; 95% CI: [0.10, 7.34], P = 0.04, I^2^ = 70%) and ≥ 12 months (MD = 3.24; 95% CI: [0.56, 5.91], P = 0.02, I^2^ = 56%) of follow-up.

**Conclusion:**

Bariatric surgery alleviated POP symptoms in women with obesity both during short-term (3–6 months) and long-term (≥ 12 months) follow-up.

**Graphical Abstract:**

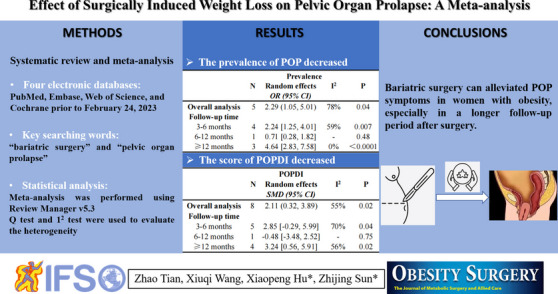

**Supplementary Information:**

The online version contains supplementary material available at 10.1007/s11695-023-06867-x.

## Introduction

With the improvement of living standards and changes in dietary habits, the number of people with obesity is increasing worldwide. Obesity is an epidemic disease that can adversely affect overall health and is associated with metabolic disorders such as diabetes, cardiovascular disease, polycystic ovary syndrome and pelvic floor disorders (PFDs) [1–3].

Pelvic organ prolapse (POP), one of the most common PFDs, is caused by the weakening of supporting structures of the pelvic floor [4]. POP is a common gynecologic disorder especially in postmenopausal women, with a prevalence of approximately 50%; although not fatal, it can seriously undermine the quality of life (QoL) of individuals [5, 6]. Obesity is a risk factor for PFD; [7, 8] increased intra-abdominal and intravesical pressure are known to be positively correlated with BMI in individuals with obesity [9]. Additionally, obesity is associated with the occurrence of diabetes mellitus and hypertension, which are also risk factors for PFD [10, 11].

Recent studies have described the effect of weight loss on PFDs symptoms in patients with obesity. Bariatric surgery (e.g., Roux-en-Y gastric bypass, sleeve gastrectomy, and laparoscopic adjustable gastric band) is an effective intervention for the treatment of obesity and related comorbidities [12, 13]. Several recent studies have demonstrated the positive effect of bariatric surgery on certain aspects of PFD such as urinary incontinence (UI) [14, 15], fecal incontinence (FI) [16], and sexual dysfunction[17]; and two recent meta-analyses also evaluated the effect of bariatric surgery on POP symptoms, while the conclusions were contradictory [18, 19].

With the rising rates of obesity among women, the demand for services to treat associated health risks is projected to increase. The prevalence of POP is also increasing continually with the aging of the human population which is a major public health concern that warrant more extensive investigation. In order to determine whether bariatric surgeries have the effect on alleviating POP symptoms, we performed a meta-analysis of published studies on POP symptoms in patients with surgically induced weight loss.

## Materials and Methods

The study protocol of this meta-analysis was registered with PROSPERO (registration no. CRD42023407714; http://www.crd.york.ac.uk/PROSPERO) and obeyed the Meta-analysis of Observational Studies in Epidemiology (MOOSE) guidelines [20].

### Data Source and Search Strategy

Four electronic databases (PubMed, Embase, Web of Science, and Cochrane) were searched for relevant studies. The final search was carried out on February 24, 2023 using the key words “bariatric surgery” and “pelvic organ prolapse”. The full search string in PubMed was as follows: (“bariatric surgery”[MeSH] OR bariatric surgery OR bariatric OR gastric bypass OR duodenal switch OR gastric balloon OR lap-band OR AspireAssist OR vBloc therapy OR metabolic surgery OR weight loss surgery) AND (“Pelvic floor disorders”[MeSH] OR “pelvic organ prolapse”[MeSH] OR “cystocele”[MeSH] OR “rectocele”[MeSH] OR “uterine prolapse”[MeSH] OR “rectal prolapse”[MeSH] OR cystocele OR rectocele OR pelvic floor disorder OR pelvic floor dysfunction OR pelvic organ prolapse OR uterine prolapse OR rectal prolapse OR POP).

### Study Selection

Inclusion criteria were as follows: i) studies evaluating the effects of bariatric surgery on POP with both pre- and postoperative data; ii) peer-reviewed study in English; and iii) no restrictions on the type of operation and follow-up time. Exclusion criteria were as follows: i) studies lacking of experimental study design (e.g., reviews, conference abstracts, case reports, etc.); ii) experiments performed on cells and animals; and iii) non-relevant or lacking data required for analysis.

### Data Screening and Extraction

Selected articles were exported to Endnote and duplicates were discarded. Two reviewers independently cataloged and organized the studies. Disagreements were resolved by consulting a third investigator. Two primary reviewers independently performed data extraction according to a predesigned form. The extracted data were first author; publication year; country; study type; age range; sample size; follow-up time; type of surgery and pre- and post-operation body mass index (BMI) and POP symptoms of participants.

### Measurement of Outcomes

In most of the studies, POP symptoms were evaluated with the Pelvic Organ Prolapse Distress Inventory 6-item (POPDI-6) and Pelvic Organ Prolapse Impact Questionnaire 7-item (POPIQ-7); these are subscales of Pelvic Floor Distress Inventory and Pelvic Floor Impact Questionnaire, respectively, which are two validated scales to assess the adverse impact of PFD on QoL [21]. The POPDI-6 has 6 questions and the POPIQ-7 has 7, each pertaining to whether a symptom exists and the degree to which it negatively affects the respondent’s QoL. Both the POPDI-6 and POPIQ-7 are scored from 0 (least distress) to 100 (greatest distress), with a higher score indicating greater symptom severity and more negative effects on QoL (Table 1).

**Table 1:** Characteristics of included studies

Study	Country	Type	Age	Pre-BMI	Post-BMI	Size	Follow	Type of surgery	Outcomes
Romero et al. 2016	USA	Cohort	48.8 ± 10.5^*^	47.5 ± 9.4^*^	32.7 ± 8.1^*^	72	6–12 m	LRYGB, LSG, LAGB	P
Cuicchi et al. 2013	Italy	Cohort	42 (20–66)^#^	43.8 ± 8.5^*^	30.0 ± 5.9^*^	87	6, 12 m	RYGB	P, POPDI, POPIQ
Leshem et al. 2017	Israel	Cohort	48.0 ± 12.0^*^	41.0 ± 4.9^*^	32.0 ± 5.2^*^	56	3–6 m	LSG, SAGB	P, POPDI
McDermott et al. 2012	USA	Cohort	47.5 ± 10.9^*^	43.7 ± 6.0^*^	29.0 ± 5.1^*^	64/63^$^	6, 12 m	LGB	P, POPDI, POPIQ
Whitcomb et al. 2012	USA	Cohort	43.3 ± 11.8^*^	39.7 ± 6.2^*^	34.0 ± 5.6^*^	98/69^$^	6, 12 m	LAP-BAND, LSG	P
Olivera et al. 2012	Israel	Cohort	41.3 ± 12.3^*^	45.8 ± 6.5^*^	-	36	3.152 y	RYGB, LSG, LAGB	POPIQ
Leshem et al. 2018	Israel	Cohort	41.6 ± 11.8^*^	41.6 ± 4.6^*^	27.5 ± 4.4^*^	18/4^$^	3–6 m, 12–24 m	LSG, SAGB	POPDI
Mazoyer et al. 2019	France	Cohort	43.0 ± 11.8^*^	41.0 ± 5.4^*^	-	72	12 m	RYGB, SG	POPDI
Wasserberg et al. 2007	USA	Cohort	45 (20–67) ^#^	45 (35–75) ^#^	28 (22–44)^#^	46	18.6 m	RYGB, Duodenal switch, SG	POPDI
Knepfler et al. 2016	France	Cohort	41.4 ± 11.4^*^	44.5 ± 6.3^*^	31.8 ± 5.8^*^	70	11.3 m	GB, SG, Gastric resection	POPDI
Shimonov et al. 2017	Israel	Cohort	41.3 ± 11.5^*^	42.0 ± 4.7^*^	33.0 ± 4.7^*^	77	6 m	LSG	POPDI

Data are presented as mean ± standard deviation^*^, median (range)^#^ and preoperative/postoperative^$^. *LAGB* laparoscopic adjustable gastric band, *LAP-BAND* laparoscopic gastric banding, *LRYGB* laparoscopic Roux-en-Y gastric bypass, *LSG* laparoscopic sleeve gastrectomy, *m* month, *P* prevalence, *POPDI* Pelvic Organ Prolapse Distress Inventory, *POPIQ* Pelvic Organ Prolapse Distress Inventory, *post-BMI* postoperative body mass index, *pre-BMI* preoperative body mass index, USA, the United States of America; y, year

### Risk of Bias Assessment

All included studies were cohort studies. The Newcastle–Ottawa Scale (NOS) [22] was used to assess study quality; all of the included studies had a score ≥ 5, and those with a score ≥ 7 were classified as being of high quality (Table 2).

**Table 2:** The quality of included studies

Study	Selection	Comparability	Outcome	NOS
Romero-Talamás et al. 2016	2	2	3	7
Cuicchi et al. 2013	2	2	3	7
Leshem et al. 2017	2	2	3	7
McDermott et al. 2012	2	2	3	7
Whitcomb et al. 2012	2	2	2	6
Olivera et al. 2012	2	2	3	7
Leshem et al. 2018	2	1	2	5
Mazoyer et al. 2019	2	2	3	7
Wasserberg et al. 2007	2	2	3	7
Knepfler et al. 2016	2	2	3	7
Shimonov et al. 2017	2	2	3	7

*NOS* the Newcastle–Ottawa Scale

### Statistical Analysis

Statistical analysis was performed using Review Manager v5.3 (https://training.cochrane.org/online-learning/core-software/revman) and Stata v12.0 (https://www.stata.com/). We calculated mean difference (MD) with 95% confidence interval (CI) for continuous variables and odds ratio (OR) with 95% CI for dichotomous variables. The chi-squared statistic and *I*^2^ tests were used to calculate heterogeneity and P < 0.05 or I^2^ > 50% was assessed as high heterogeneity [23]. When heterogeneity was high, a random-effects model was used and sensitivity analysis was conducted to evaluate the robustness of the results (Supplementary Fig. 1). Begg’s test[24] was used to evaluate potential publication bias.

## Results

### Literature Search

**Fig. 1:**
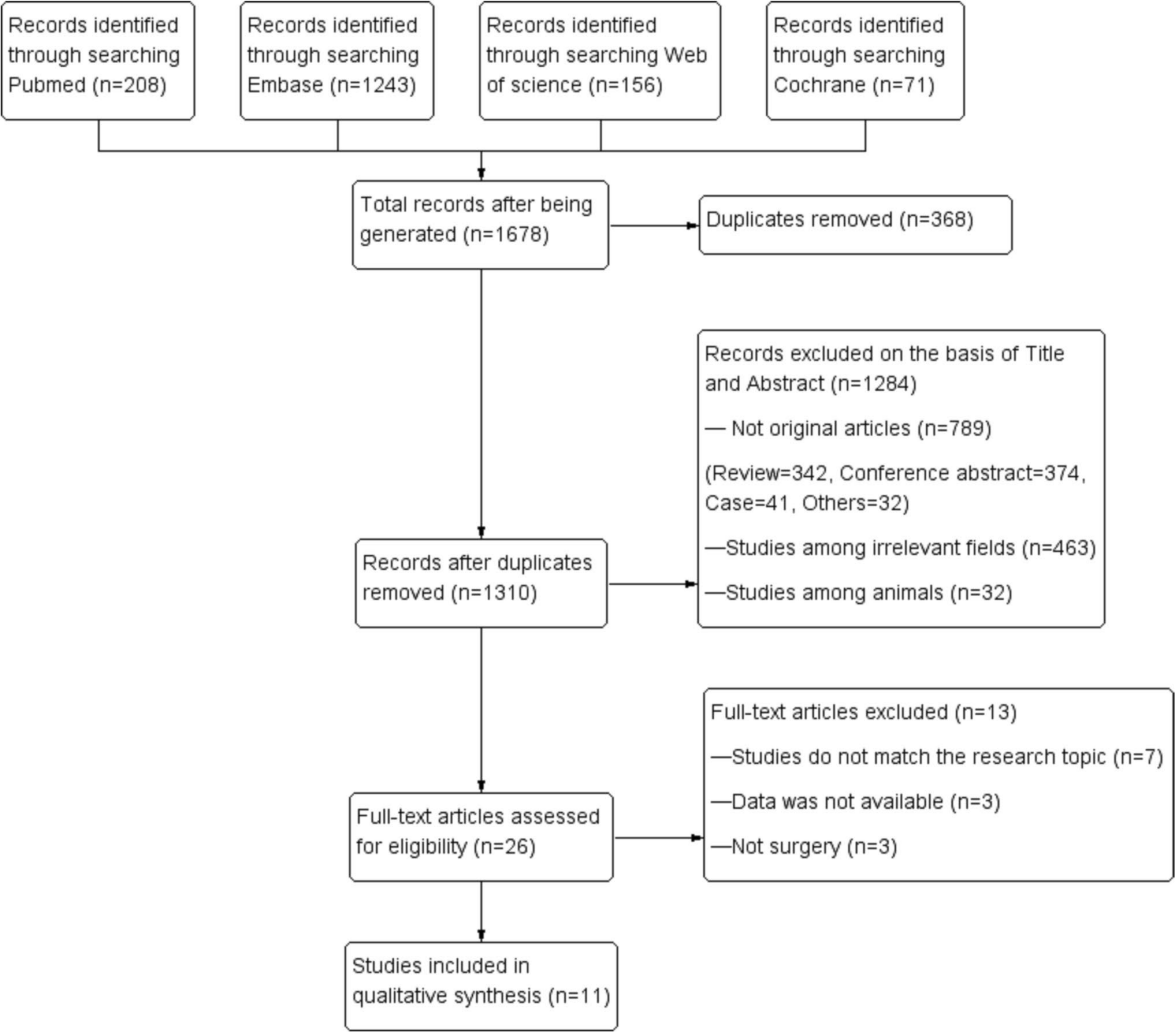
Flow chart of literature search

The literature search strategy is illustrated in Fig. 1 and 11 studies [25–35] were ultimately included in the meta-analysis.

### Characteristics and Quality of Included Studies

The characteristics of the included studies are summarized in Table 1. In total, there were 696 participants in the 11 included studies, which all had a prospective cohort design. Four studies were conducted in the United States [25, 28, 29, 33], 3 in Israel [27, 30, 31, 35], 2 in France [32, 34], and 1 in Italy [26]. Five studies focused on the prevalence of POP [25–29], of which 3 also reported changes in POPDI-6 [26–28] and 2 also reported POPIQ-7 (n = 2)[26, 28] scores; the remaining 6 studies reported changes in POPDI-6 (n = 5)[31–35] or POPIQ-7 (n = 1)[30] scores. Of the 5 studies that examined the prevalence of POP, symptomatic POP was self-reported in 4 [26–29]; in 1 study, POP was also objectively analyzed with the Pelvic Organ Prolapse Quantification System[25] in addition to being self-reported. Regarding postoperative follow-up time, 4 studies reported outcomes at 6 months [26, 28, 29, 35], 3 at 12 months [26, 28, 29], 2 at 3–6 months [27, 31], 1 at 6–12 months [25], and 1 at 12–24 months [31]; and 3 studies had a median follow-up time of 11.3 months [34], 18.6 months [33], and 3.152 years [30]. To facilitate the subgroup analysis, we divided the follow-up time into 3–6 months [26–29, 31, 35], 6–12 months (excluding 6 and 12 months) [25, 34], and ≥ 12 months [26, 28–30, 33] (Table 3). Study quality according to the NOS was high for 9 studies [25–28, 30, 32–37] and moderate for 2 studies [29, 31] (Table 2).

**Table 3:** Subgroup analysis of POP prevalence and POPDI score before and after bariatric surgery based on follow-up time, study quality and region

	Prevalence				POPDI				
	N	Random effects *OR (95% CI)*	I^2^	P		N	Random effects *SMD (95% CI)*	I^2^	P
Overall analysis	5	2.29 (1.05, 5.01)	78%	0.04		8	2.11 (0.32, 3.89)	55%	0.02
Follow-up time									
3–6 months	4	2.24 [1.25, 4.01]	59%	0.007		5	3.72 [0.10, 7.34]	70%	0.04
6–12 months	1	0.71 [0.28, 1.82]	-	0.48		1	-0.48 [-3.48, 2.52]	-	0.75
≥ 12 months	3	4.64 [2.83, 7.58]	0%	< 0.0001		4	3.24 [0.56, 5.91]	56%	0.02
Study Quality									
*High*	4	2.19 (0.93, 5.13)	83%	0.07		7	2.03 (0.22, 3.83)	59%	0.03
*Moderate*	1	3.66 (0.42, 32.01)	-	0.24		1	10.1 (-7. 4, 27.6)	-	0.26
Region									
*USA*	3	2.07 (0.54, 7.92)	77%	0.29		1	1.81 (0.70, 2.92)	-	0.001
*France*	0	-	-	-		2	0.41 (-1.93, 2.76)	0%	0.73
*Israel*	1	1.36 (0.81, 2.29)	-	0.24		4	2.12 (-1.65, 5.89)	56%	0.27
*Italy*	1	5.21 (2.62, 10.36)	-	< 0.0001		1	6.50 (2.93,10.07)	-	0.0004

*CI* confidence interval, *OR* odds ratio, *POP* pelvic organ prolapse, *POPDI* Pelvic Organ Prolapse Distress Inventory, *SMD* standardized mean difference, *USA* United States of America

### Primary Results

**Fig. 2:**
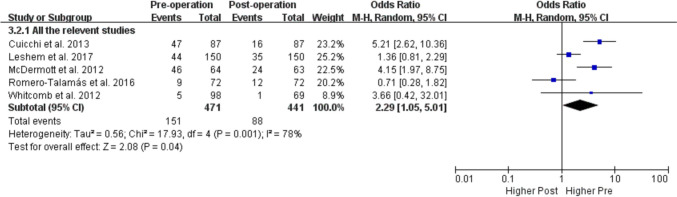
Meta-analysis comparing POP prevalence before and after bariatric surgery

**Fig. 3:**
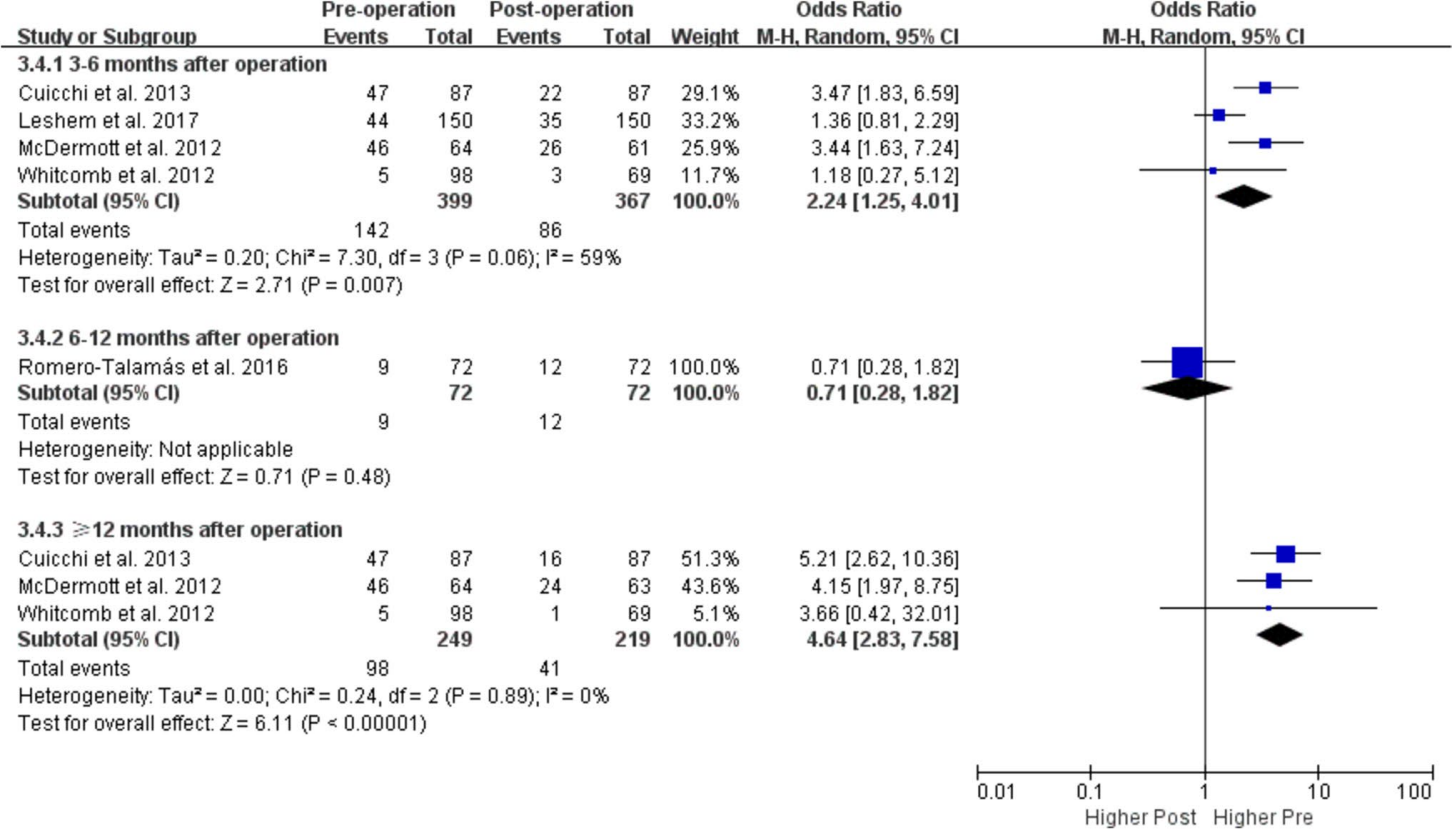
Meta-analysis comparing POPDI-6 scores before and after bariatric surgery

As shown in Fig. 2, the prevalence of POP symptoms decreased significantly after bariatric surgery. And POPDI-6 scores also decreased after the surgery which indicates an improvement in POP symptoms (Fig. 3).

### Secondary Results

**Fig. 4:**
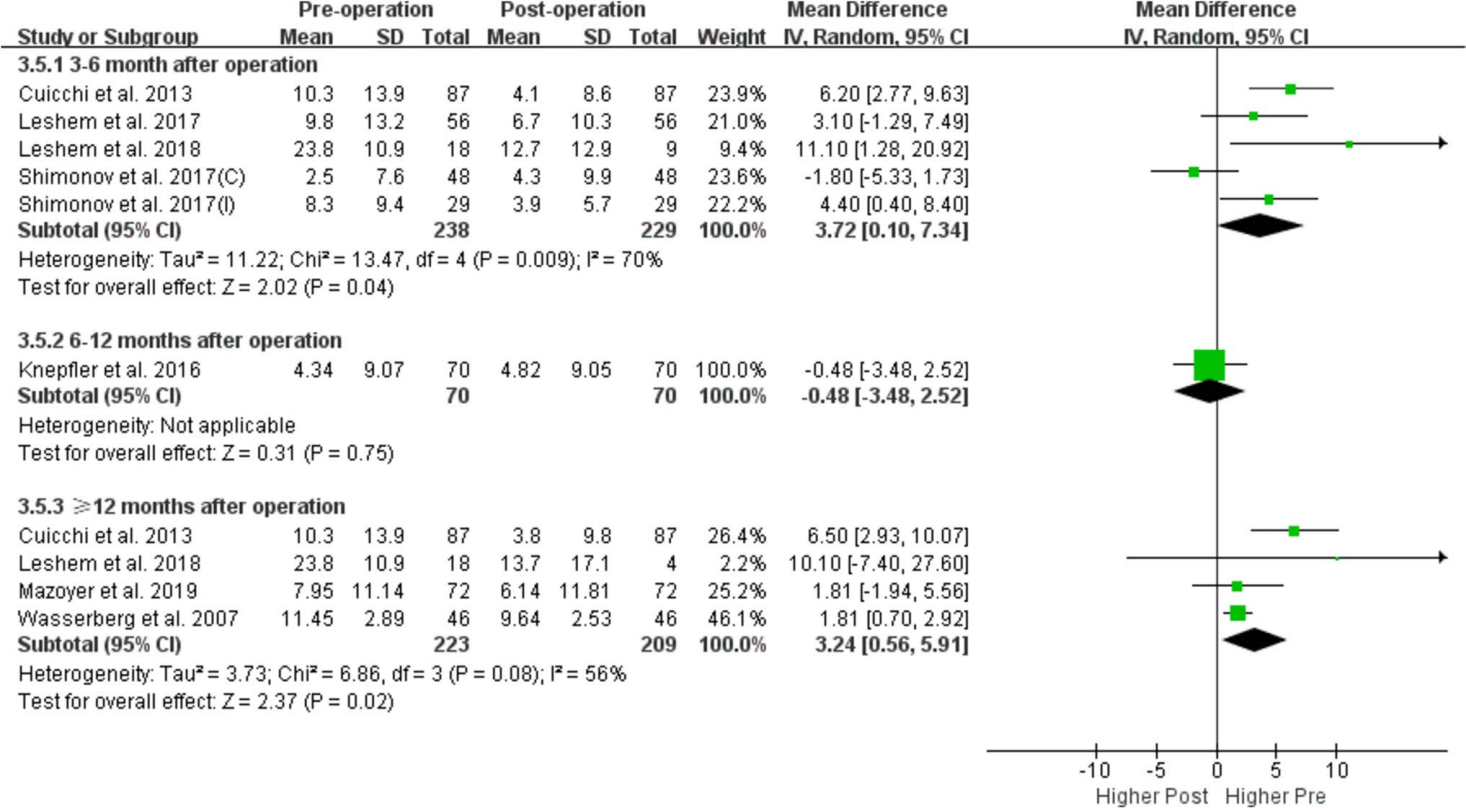
Subgroup-analysis comparing POP prevalence based on follow-up time (3–6 months, 6–12 months and 12 month after bariatric surgery)

**Fig. 5:**
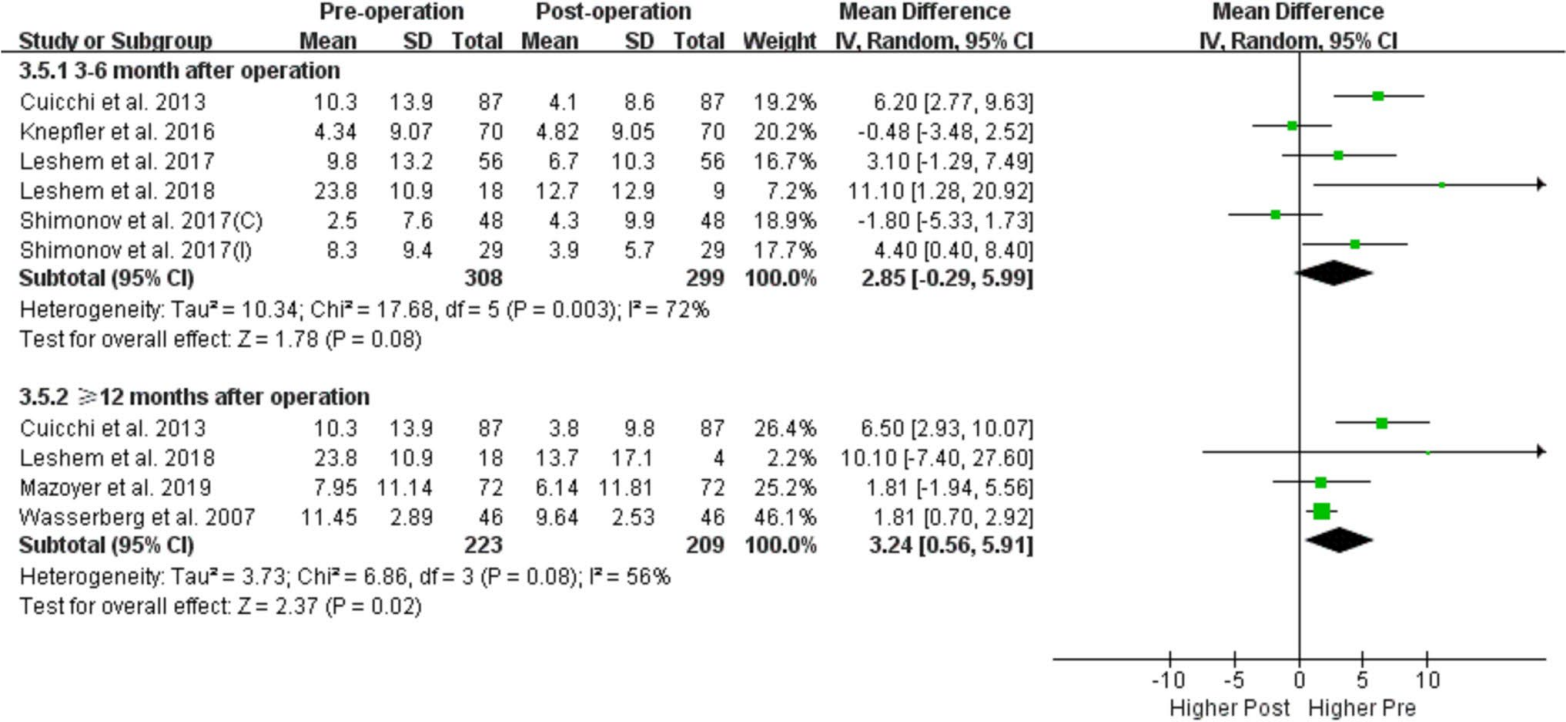
Subgroup-analysis comparing POPDI-6 scores based on follow-up time (3–6 months, 6–12 months and ≥ 12 month after bariatric surgery)

Subgroup analyses according to follow-up time, study region, and study quality failed to detect the source of heterogeneity (Table 3). The prevalence of POP was decreased both at 3–6 months and 12 months of follow-up compared to before the surgery (Fig. 4) and the POPDI-6 scores also decreased after bariatric surgery both at 3–6 months and ≥ 12 months of follow-up (Fig. 5). Only 2 studies reported the change in POP prevalence and POPDI-6 scores at 6–12 months after surgery, and no significant difference was found compared with pre surgery (Figs. 4 and 5)**.**

### Publication Bias

Publication bias was assessed with Begg’s funnel plots and no publication bias was found (Supplementary Fig. 2).

## Discussion

There is accumulating evidence of a link between obesity and PFDs, which significantly impact the QoL of patients and thereby impose a substantial social and economic burden. Many previous studies have demonstrated an improvement in PFDs after bariatric surgery, but the effects of this intervention on POP—a common type of PFD—remain unclear. In this meta-analysis, we demonstrated that the prevalence of POP and POPDI-6 scores were decreased after bariatric surgery, providing evidence for the clinical benefits of this intervention for POP.

Bariatric surgery has been shown to positively impact weight loss and some aspects of PFDs such as UI [14, 15], FI [16], and sexual dysfunction [17]. However, the effects of bariatric surgery on POP are controversial. Consistent with our results (Fig. 2), one meta-analysis of 4 relevant studies reported that POPDI-6 scores were significantly decreased after bariatric surgery [19]. However, Montenegro et al. [18] compared the prevalence of POP before versus after bariatric surgery based on the same 5 studies in their meta-analysis and reached conclusions that differed from ours (Fig. 1). A possible reason for the discrepancy is the selection of data from one of the studies [29]; Montenegro et al. [18] used 6 months post-surgery data from this study although the total follow-up time was up to 12 months [29]. We pooled the 12-month data for our meta-analysis, which is more reasonable. Moreover, the results of the subgroup analysis by follow-up time showed that the prevalence of POP was decreased both at 3–6 and 12 months after bariatric surgery, supporting the robustness of our results (Fig. 4).

As the variable follow-up time across studies are bound to have an impact on the accuracy of the results, we performed a subgroup analysis based on the duration of follow-up to increase statistical power for the first time. We found that both POP prevalence and POPDI-6 scores decreased at 3–6 and ≥ 12 months of follow-up (Figs. 4 and 5). A caveat is that the follow-up time of the included studies was relatively short, with just 3 studies having a follow-up time more than 1 year (1–2 years [31] and a mean follow-up of 18.6 months [33] and 3.152 years [30]). Additionally, we performed subgroup analyses by study quality and region (Table 3). We found that POPDI-7 scores in high-quality studies were consistent with those in the overall analysis whereas POP prevalence showed no significance. The discrepancy may be related to the smaller number of included studies and higher heterogeneity, but may also indicate the instability of the current results. In the subgroup analysis by region, we found that 3 studies with data for POP prevalence in the US and 4 studies with data for POPDI scores from Israel showed different results from the overall analysis, which needs to be validated in future work. In addition, one of the included studies found that in obese patients with symptoms of UI, these symptoms along with POP were significantly improved after bariatric surgery, whereas no improvement in POP symptoms was observed after bariatric surgery in patients without UI symptoms, indicating that the improvement in PFD symptoms after this intervention is more obvious in obese women with multiple PFD symptoms.

This meta-analysis was subject to several limitations. First, the number of relevant studies is limited and some variables showed heterogeneity and instability across studies. Second, the diagnosis of POP was mainly made based on self-reported symptoms, and there was no definitive diagnosis that was independently confirmed. Third, there were variations in the surgical techniques used and phenotypic characterization of obesity across studies, with relatively short follow-up periods in some cases. Fourth, all of the included studies had a nonrandomized cohort design; as such, the risk of selection bias was unavoidable. Finally, the analysis examined the effect of bariatric surgery on POP without taking into account the potential complications associated with this procedure.

## Conclusion

This meta-analysis provides evidence that bariatric surgery alleviated POP symptoms in women with obesity both during short-term (3–6 months) and long-term (≥ 12 months) follow-up. However, due to the limitations of the number and quality of the current studies, further randomized controlled trials on a larger scale and of longer duration are needed to validate these findings.

### Supplementary Information

Below is the link to the electronic supplementary material.

Supplementary file1 (TIF 1526 KB)

Supplementary file2 (TIF 1071 KB)

Supplementary file3 (PDF 37 KB)

## Data Availability

Requests for reprints should be directed to the corresponding authors.
