# Supplementary figures and images for: Effect of Surgically Induced Weight Loss on Pelvic Organ Prolapse: A Meta-analysis
Source: Obes Surg. 2023 Oct 7;33(11):3402–10. doi: 10.1007/s11695-023-06867-x (PMC10602998; doi:10.1007/s11695-023-06867-x)

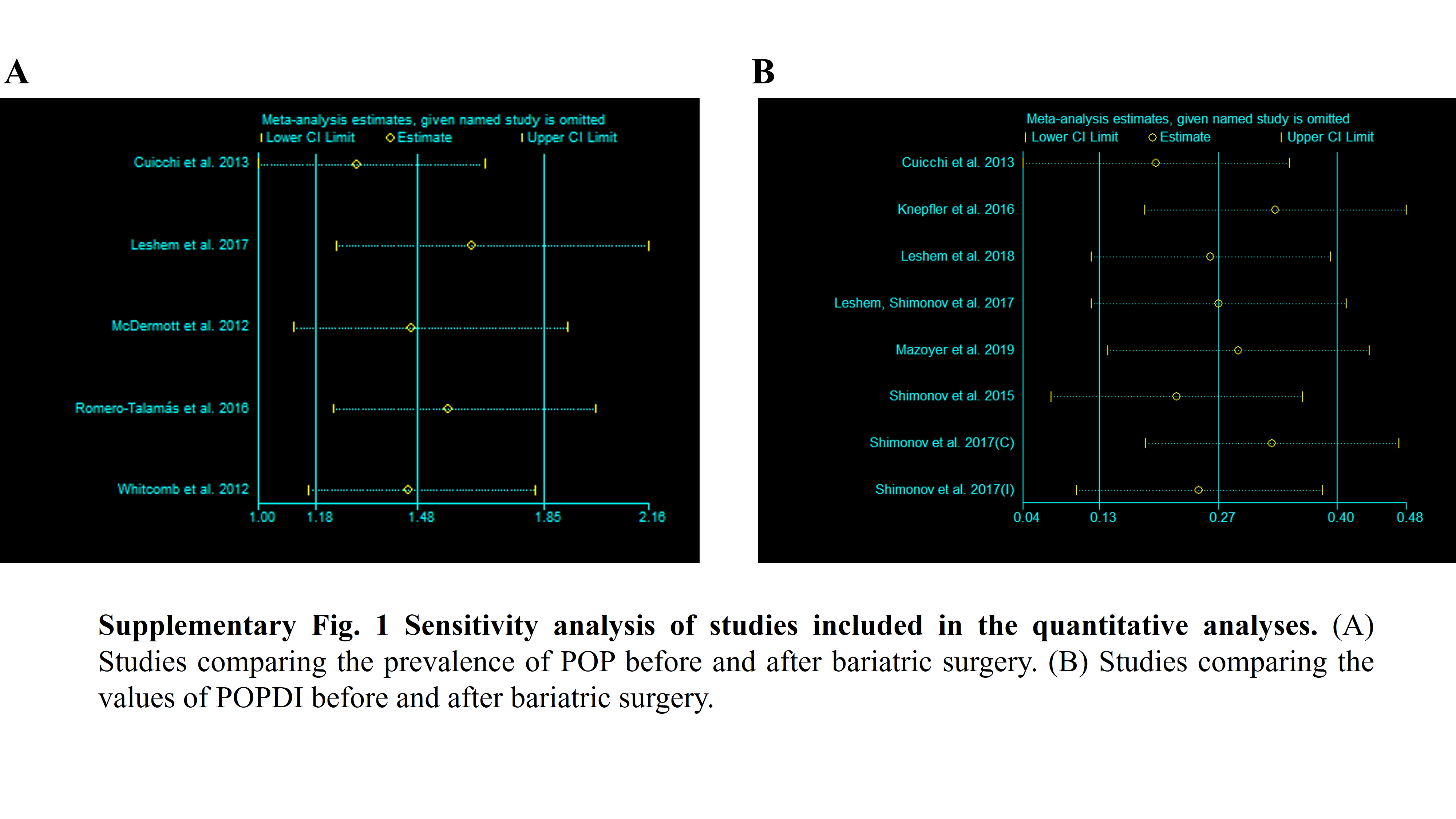

Supplement: Supplementary file 1 — Supplementary file1 (TIF 1526 KB) [file 11695_2023_6867_MOESM1_ESM.tif]

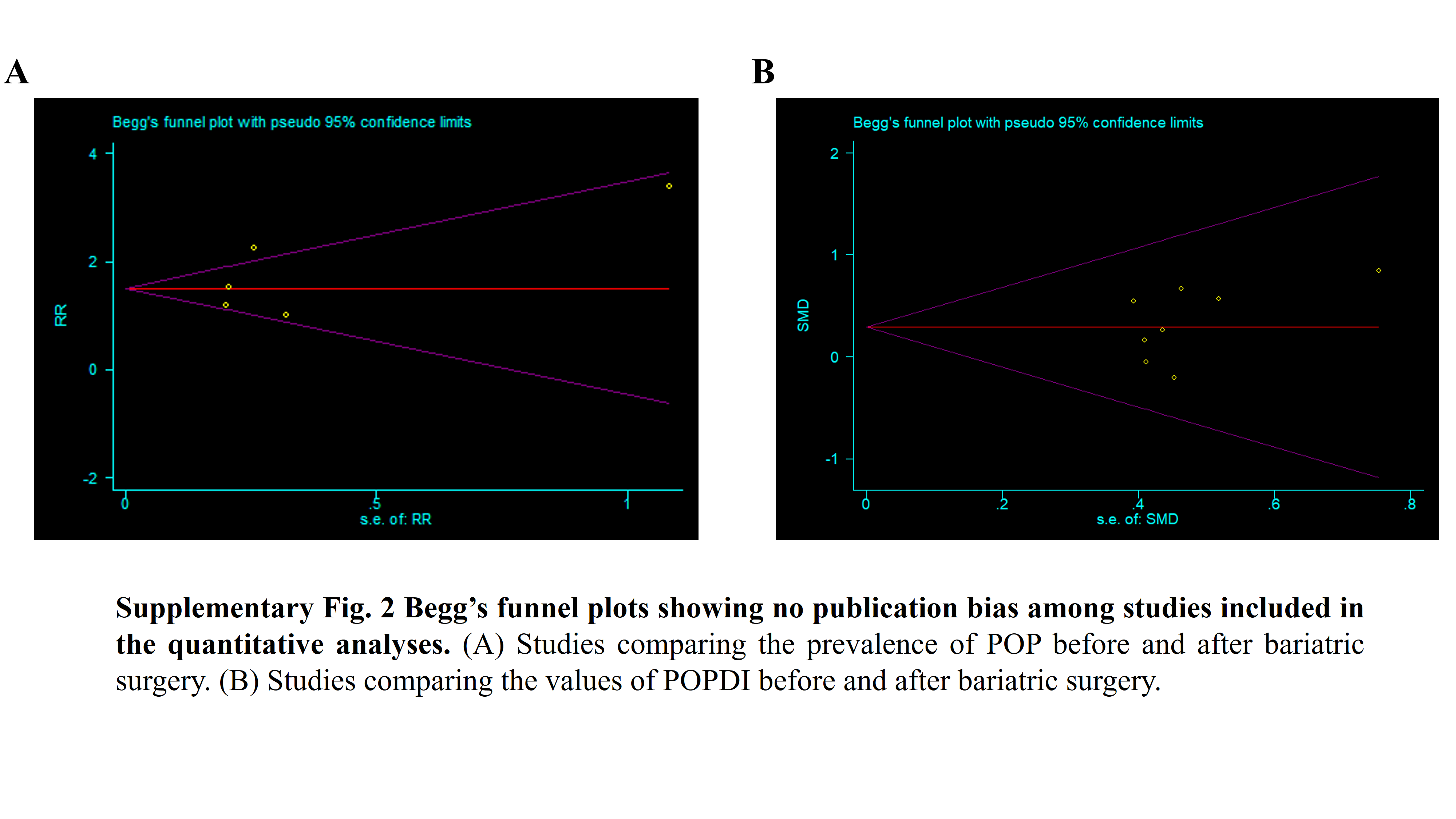

Supplement: Supplementary file 2 — Supplementary file2 (TIF 1071 KB) [file 11695_2023_6867_MOESM2_ESM.tif]
