# Supplementary material for: Effect of Surgically Induced Weight Loss on Pelvic Organ Prolapse: A Meta-analysis
Source: Obes Surg. 2023 Oct 7;33(11):3402–10. doi: 10.1007/s11695-023-06867-x (PMC10602998; doi:10.1007/s11695-023-06867-x)
Supplement: Supplementary file 3 — Supplementary file3 (PDF 37 KB) [file 11695_2023_6867_MOESM3_ESM.pdf]

| | | Pre | | Post | |
|---|---|---|---|---|---|
| | | m1 | n1 | m2 | n2 |
| POP 3-6 | Cuicchi et al. 2013 | 47 | 87 | 22 | 87 |
| | Leshem et al. 2017 | 44 | 150 | 35 | 150 |
| | McDermott et al. 2012 | 46 | 64 | 26 | 61 |
| | Whitcomb et al. 2012 | 5 | 98 | 3 | 69 |
| POP 6-12 | Romero-Talamás et al. 2016 | 9 | 72 | 12 | 72 |
| POP≥12 | Cuicchi et al. 2013 | 47 | 87 | 16 | 87 |
| | McDermott et al. 2012 | 46 | 64 | 24 | 63 |
| | Whitcomb et al. 2012 | 5 | 98 | 1 | 69 |

| | | Pre | | | Post | | |
|---|---|---|---|---|---|---|---|
| | | m1 | sd1 | n1 | m2 | sd2 | n2 |
| PPODI 3-6 | Cuicchi et al. 2013 | 10.3 | 13.9 | 87 | 4.1 | 8.6 | 87 |
| | Leshem et al. 2017 | 9.8 | 13.2 | 56 | 6.7 | 10.3 | 56 |
| | Leshem et al. 2018 | 23.8 | 10.9 | 18 | 12.7 | 12.9 | 9 |
| | Shimonov et al. 2017(C) | 2.5 | 7.6 | 48 | 4.3 | 9.9 | 48 |
| | Shimonov et al. 2017(I) | 8.3 | 9.4 | 29 | 3.9 | 5.7 | 29 |
| POPDI 6-12 | Knepfler et al. 2016 | 4.34 | 9.07 | 70 | 4.82 | 9.05 | 70 |
| POPDI ≥12 | Cuicchi et al. 2013 | 10.3 | 13.9 | 87 | 3.8 | 9.8 | 87 |
| | Leshem et al. 2018 | 23.8 | 10.9 | 18 | 13.7 | 17.1 | 4 |
| | Mazoyer et al. 2019 | 7.95 | 11.14 | 72 | 6.14 | 11.81 | 72 |
| | Wasserberg et al. 2007 | 11.45 | 2.89 | 46 | 9.64 | 2.53 | 46 |
